# Surveying the utilization of cold plasma and plasma-activated water on food pigments, bioactive compounds, enzymes, vitamins, fatty acid, and essential oils: Considerations, mechanisms, and future trends

**DOI:** 10.1016/j.fochx.2025.102509

**Published:** 2025-05-11

**Authors:** Shiva Vedaei, Arash Dara

**Affiliations:** a Department of Food Science and Technology, Shahreza Branch, Islamic Azad University, Shahreza, Iran; b Department of Green Technologies in Food Production and Processing, Research Institute of Food Science and Technology (RIFST), Mashhad, Iran

**Keywords:** Cold plasma, Bioactive compounds, Pigments, Enzymes, Fatty acid, Essential oils

## Abstract

Cold plasma (CP) technology has emerged as a novel, non-thermal, and eco-friendly method for enhancing food processing while reducing nutrient loss and degradation compared to traditional methods. This review collates the latest information on the effects of cold plasma's reactive species on food constituents, including bioactive compounds, pigments (chlorophyll, betalain, carotenoids, anthocyanins), enzymes, vitamins, fatty acid profiles, and essential oils. It also explores the mechanisms and functional properties of various cold plasma types (dielectric barrier discharge, jet plasma) and plasma-activated water, highlighting key factors like time, voltage, frequency, and gas to enhance the technology's efficiency. Even so, a thorough insight into the impact of cold plasma on food constituents and the underlying interaction mechanisms remains essential. Understanding the limitations of cold plasma treatment is crucial for advancing the recognition and acceptance of this modern technology.

## Highlights

• Insight into the impact of CP on food constitutes and their mechanisms.
• CP and PAW were beneficial for the stability of bioactive compounds.
• The reaction including improvement and degradation that occur in each component.
• Application of Cold plasma on enzyme inactivation such as PPO and POX.
• The efficiency of CP on food pigments including anthocyanins and others.

## Graphical abstract

**Figure:**
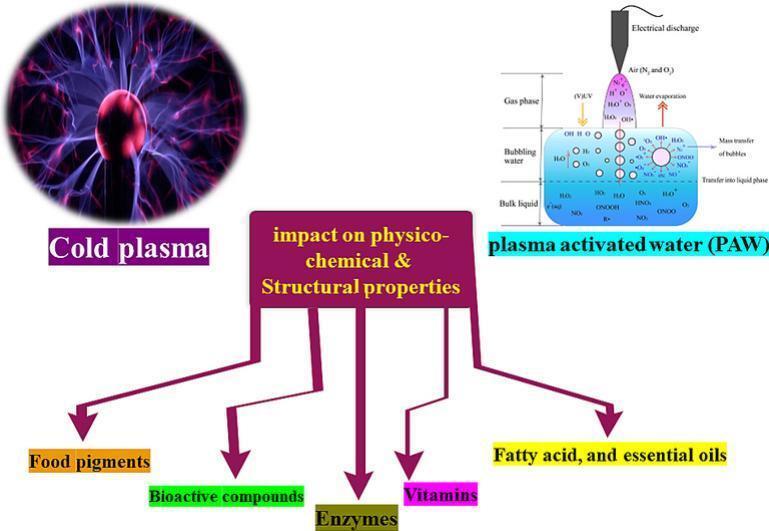

## 1 Introduction

Foods processed with non-thermal processing have the most potential to increase functional properties, shelf life, and freshness. In recent years, consumers have become more interested in buying food with high levels of nutritional properties, including bioactive compounds, essential oils, fatty acid profiles, vitamins, and minerals. Many studies confirmed that heat treatment for food preparation could impact food quality, such as significantly dropping the nutritional value, and deteriorating texture, flavor, and color (Harikrishna et al., 2023). Cold plasma as non-thermal processing is one of the breakthrough technologies with a wide range of benefits in food processing channels and is known as an innovative technology (Kaavya et al., 2023). This technology has been utilized in the food industry to deactivate enzymes and microorganisms, enhance bioactive compounds, and boost antioxidant activity (Laroque et al., 2022). Generally, plasma is divided into hot plasma (thermal) and cold plasma (non-thermal or non-isothermic, low-temperature), cold plasma is suitable for treating heat-sensitive foods as well as an environmental friend treatment (Bezerra et al., 2023). Cold plasma utilizing ionized gas with free radicals, electrons, ions, and uncharged particles, has gained significant attention. One of the most important characteristics of non-equilibrium cold plasma is the capability of mixing reactive oxygen species (ROS) and reactive nitrogen species (RNS) at ambient temperature (Jaddu et al., 2024). It is employed for bacteria and virus inactivation, food product treatments, and surface modifications. Cold plasma can also deactivate enzymes and enhance the antioxidant levels of food products (Gao et al., 2022). Emerging technologies effectively deactivate enzymes involved in browning fruits and vegetables, such as polyphenol oxidase (PPO) and peroxidase (POX) (Kumar, Pipliya, Srivastav, & Srivastava, 2024; Paixão et al., 2019; Zhao et al., 2024), while also enhancing levels of vitamins C, A, and B in numerous studies (Fernandes et al., 2019; Ji et al., 2020; Kumar, Pipliya, Srivastav, Srivastava, Battula, & Sen, 2024; Paixão et al., 2019; Zhou et al., 2023). In addition, cold plasma treatment improved the total bioactive compounds in Mango (Yanclo et al., 2024), green tea (Keshavarzi et al., 2020), strawberry (Rana et al., 2020), and pomegranate juice (Herceg et al., 2016). Also, utilizing atmospheric cold plasma to pretreat grape processing by-products improved the recovery of bioactive compounds by creating a hydrophilic surface and disrupting cell structure in grape pomace (Bao et al., 2020). However, the higher level of plasma treatment can accelerate the degradation of phenolic compounds because it breaks the aliphatic chain and benzene rings (Jaddu et al., 2024; Sruthi et al., 2022). On the other hand, cold plasma treatment could increase the essential oils (EOs) components and optimize the EOs yield extraction (Gunathilake et al., 2025; Rezaei et al., 2021; Shokoohi et al., 2024). These components are famous for their beneficial effects, including antioxidant, anti-inflammatory, and anti-cancer properties (Khan et al., 2024). In addition, food pigments such as anthocyanins, carotenoids, betalain, and chlorophyll could affected by cold plasma. This innovative technology has led to an increase in anthocyanins in blueberries (Ji et al., 2020; Pathak et al., 2020; Zhou et al., 2023).

Consequently, this review aims to present: 1. the effects of various types of cold plasma and Plasma activated water in the food constituents, including bioactive compounds, natural and synthesized food pigments (chlorophyll, betalain, carotenoids, anthocyanins), enzymes, vitamins, fatty acid profiles, and essential oils. 2. Their applications in food process engineering, 3. Beneficial efficiency of cold plasma and plasma activated water on structuring, and food matrix with emphasis on usability in food formulations, and finally 4. Review some limitations and challenges that will be faced in the food industry.

## 2 Cold plasma

Cold plasma is defined as a novel method in food processing. Nonetheless, further research is needed on the interaction mechanism between reactive species and bioactive compounds in various processing (plasma generation) and matrix effects. Various types of cold plasma exist, including dielectric barrier discharge (DBD) plasma, jet plasma, glow plasma, gliding arc plasma, arc plasma, microwave-driven discharge, radio frequency-driven discharge, and Corona discharge (Fernandes et al., 2019; Kumar et al., 2023). Cold plasma exhibits both positive and negative effects on different compounds, influenced by factors such as process gas, plasma source, treatment duration, input voltage, and food matrix composition (Sruthi et al., 2022). Different gases, like oxygen (O), argon (Ar), nitrogen (N), helium (He), and atmospheric air, when subjected to an electric discharge, create positive and negatively charged ions, electrons, and charged particles in cold plasma (Alves Filho et al., 2020).

## 3 The effect of cold plasma on the food matrix

The application of cold plasma to various foods has been extensively researched, as understanding its mechanisms is crucial for using plasma as a food processing aid (Sruthi et al., 2022). Phenolic compounds protect cell tissues from harmful species like singlet oxygen, superoxide, peroxide radicals, hydroxyl radicals, and peroxynitrite. These compounds are bound to cell membranes, requiring energy to release total phenols. Cold plasma technology is at the forefront of this purpose. The breakdown of covalent bonds and cell membranes is possibly due to the activation of enzymes, chemically reactive species, charged particles, and UV photons produced from cold plasma (Hou et al., 2019; Rodríguez et al., 2017). One key enzyme involved in phenolic compound synthesis is phenylalanine ammonia-lyase (PAL), which can be activated by cold plasma treatment. This enzyme breaks down cell walls, leading to an increase in phenolic compounds. Cold plasma processing demonstrates a synergistic effect attributed to reactive species. Some reactive species hydroxylate benzene rings in phenolic compounds, producing hydroxycyclo carbonyl radicals that reduce total phenolic acid content. Additionally, time plays a critical role in phenolic compounds because of exposure time and some oxidative or polymerization reactions. For instance, TPC in blueberries were increased after 1 min treatment and decreased in longer plasma treatment (Abouelenein et al., 2022; Astorga et al., 2022; Sarangapani et al., 2017).

Research on apple slices treated with a plasma jet (Argon gas) found that the increased hardness is due to the inactivation of enzymes responsible for softening and cell wall breakdown during ripening (Jihad et al., 2024). It may relate to direct exposure to argon plasma and the degradation of COOH and CNH2 groups in L-alanine of amino acids (Simić et al., 2007).

Loureiro et al. (2021) found that exposing tucumã to cold plasma (DBD) increases the drying rate and reduces drying time while enhancing phenolic and antioxidant compounds (Table 1) (Loureiro et al., 2021). Similarly, Batista et al. (2021) demonstrated that treating avocado pulp with cold plasma with and without lime extract effectively maintained quality and increased bioactive content (Batista et al., 2021). Also, studies in dried peppermint (Mentha piperita L.) (Kashfi et al., 2020) and strawberry fruit (Rana et al., 2020) have demonstrated an increase in total phenolic content and antioxidant activity. In contrast, Pankaj et al. (2017) reported different results for White Grape juice treated with high voltage atmospheric cold plasma (HVACP) (Pankaj et al., 2017). Furthermore, several studies have noted significant decreases in enzymatic activity in treated Pink Lady apples (Tappi et al., 2018), avocado pulp (Batista et al., 2021), and açaí pulp (Dantas et al., 2021).

**Table 1:** Effect of cold plasma on food matrix.

Food matrix	Plasma source	Experimental condition		Exposure	Process effects	Reference
		Power/Voltage	Frequency	Time		
Apple slices	Plasma jet (pressure: 5 atm)	14 kV	**–**	3–6 min	• The moisture content increased Drying processes increased Hardness(3 min) increased	Jihad et al., 2024
Tucuma	DBD	20 kV	500 and 800 Hz	10 min	• Drying rate increased Phenolic compounds and antioxidant activity increased Carotenoids decreased	Loureiro et al., 2021
Açai pulp	ACP (DBD)	20 kV	500 Hz/50 Hz	5 min/10 min	• POD and PPO decreased Phenolic content (500 Hz/5 min) increased DPPH and ORAC assays (50 Hz/10 min) increased Catechin, epicatechin, epigallocatechin, gallate, procyanidin B1, rutin, caffeic acid, chlorogenic acid (50 Hz/10 min) increased	Dantas et al., 2021
Avocado pulp	DBD	**–**	**–**	**–**	• The phenolic (gas flow 20 mL/30 in PL) increased Carotenoid (gas flow 10 mL/10 min gas flow in PW) increased Fatty acid profile, − Miristic and palmitic acids decreased - the oleic, linoleic and linolenic increased POD and PPO activities (gas flow 20 mL/20 min applied in PL) decreased ORAC-L (gas flow 10 mL/10 min in PL) increased	Batista et al., 2021
Strawberry fruit	ACP (DBD)	60 kV	50 Hz	15 min	• Chlorogenic acid, hyprin, phloretin, vanillin, gallic acid, 4-hydroxybenzaldehyde and rutin increased The total phenolic content and antioxidant activity increased	Rana et al., 2020
Dried peppermint (Mentha piperita L.)	Radio-frequency (LPCP)	50 W	13.56 MHz	20 min	• The total phenolic content and antioxidant activity (DPPH and FRAP) increased	Kashfi et al., 2020
Pink Lady apples	DBD	150 W	12.7 kHz	10–30 min	• enzymatic activity (30 min) decreased the phenolic profile (10 min) increased	Tappi et al., 2018
White Grape juice	HVACP (DBD)	80 kV	**–**	4 min	• Total phenolics, flavonoids, DPPH free radical scavenging and antioxidant capacity decreased	Pankaj et al., 2017

## 4 Cold plasma effects on bioactive compounds

Bioactive compounds, which offer great potential benefits to animals and humans, are known as secondary metabolites. These compounds can be essential or non-essential, such as polyphenols and vitamins. They serve as potent antioxidants, effectively combating free radicals and removing reactive oxygen species, thereby helping to prevent human disorders like microbial infections and cancer. Bioactive compounds are recognized for various biological activities, including enzyme inhibition, antimicrobial properties, anti-allergic effects, anticancer potential, antifouling properties, anti-inflammatory attributes, and antioxidative benefits. These compounds can be found in plants, algae, fungi, bacteria, and invertebrates. Bioactive compounds, derived from plants, are a common source of Phenolic compounds, plant sterols, organosulfur compounds, lycopene, dietary fibers, monoterpenes, and isothiocyanates (Kumar & Naraian, 2019). Phenolic compounds are derived from the phenylpropanoid metabolism of plants, involving the shikimic acid and malonic acid pathways. They are chemically classified into two main groups: phenols and polyphenols, depending on the presence of an aromatic ring bound to one or more hydroxyl groups (Nemli et al., 2024).

The structure of polyphenols is divided into flavonoids and non-flavonoids. Flavonoids encompass flavones, flavanones, isoflavones, flavanols, and anthocyanidins, while non-flavonoids include phenolic acids, stilbenes, and lignans (Abd El-Hack et al., 2023). Polyphenols consist of a benzene ring and multiple hydroxyl groups. A significant plant flavonoid found in fruits and vegetables is quercetin, which contains hydrophilic hydroxyl groups and a hydrophobic ring. Phenolic hydroxyl groups inhibit free radical chain reactions, giving quercetin antioxidant and bacteriostatic properties that play crucial roles in boosting immune function, reducing inflammation, dilating blood vessels, and more. Polyphenols like phytochemicals have been associated with lowering the risk of coronary heart disease and regulating gene expression (Gao et al., 2022). However, polyphenols face challenges such as oxidation in neutral and alkaline environments, which can restrict their physicochemical and molecular properties (Ke et al., 2023). The interaction mechanism varies between plasma reactive species and food components for each functional and bioactive component. Due to the dynamic nature of plasma species and different mechanisms for each functional and bioactive component, studying the interaction mechanism between cold plasma and bioactive compounds plays a pivotal role in enabling it to function as a food processing aid. As a result, the application of cold plasma on bioactive compounds has been researched The green tea treatment with nitrogen DBD cold plasma has shown an increase in gallic acid and catechin, as well as a decrease in epicatechin and epigallocatechin gallate (EGCG) (Keshavarzi et al., 2020). This was due to the degradation of EGCG to gallic acid during the CP treatment (Abouelenein et al., 2022). The açaí pulp was treated by an Atmospheric cold plasma (DBD) system, the epicatechin gallate, which observed an increase at 50 Hz both in 10 and 15 min, but with more time, it improved further (Dantas et al., 2021).

In assessing the effects of cold plasma on fresh-cut apples, the phenolic content increased due to catechin polymerization products as well as a rise in hydroxycinnamic acids, and chalcones (Sruthi et al., 2022; Tappi et al., 2018). Research by Kovačević, Kljusurić, et al. (2016) showed that cold plasma treatment of pomegranate and chokeberry juices increases hydroxycinnamic acids by facilitating slow reactions with plasma-generated radicals (Herceg et al., 2016; Kovačević, Kljusurić, et al., 2016).

The study of açaí pulp revealed that the levels of caffeic acid and p-coumaric acid increased with DBD cold plasma treatment at 50 Hz/10 min (Dantas et al., 2021). The rise in reactive oxygen species in the fruit pulp prompted the activation of the phenylpropanoid pathway, leading to the production of caffeic acid and p-coumaric acid. Furthermore, the reaction of p-coumaric acid with hydroxyl radicals was found to boost caffeic acid levels. The increase in these two compounds indicated the activation of PAL, Cinnamic acid 4-hydroxylase (C4H), and 4-coumarate coenzyme A ligase (C3H) enzymes in the phenylpropanoid pathway (Dantas et al., 2021; Liu et al., 2018).

Herceg et al. (2016) reported that cold plasma treatment increased the concentrations of ellagic acid. Cold plasma generates chemically reactive species, charged particles, and UV photons, which can break covalent bonds and trigger various chemical reactions (Herceg et al., 2016). This leads to enhanced cell membrane breakdown, resulting in improved hydrolysis and depolymerization. The rise in ellagic acid content may stem from the hydrolysis and depolymerization of ellagitannins, a finding also observed in strawberries (Theocharis & Andlauer, 2013) through microwave energy. Also, the increased levels of chlorogenic acid, ferulic acid, and catechin in pomegranate juice result from reactive species of plasma that can affect cell membrane breakdown, hydrolysis, and depolymerization.

Keshavarzi et al. (2020) found that the total phenolic content (TPC) in green tea increased during treatment (Keshavarzi et al., 2020), aligning with findings in pomegranate juice (Herceg et al., 2016) and Pink Lady apples (Tappi et al., 2018). In green tea (Keshavarzi et al., 2020), phenolic compounds are bound to cell membranes and require energy to release. Ion bombardment ruptured these membrane-bound compounds in the CP, aiding their extraction. Yanclo et al. (2024) reported that CP pretreatment enhances the drying rate of mango slices, total phenolic content, and antioxidant activity (Yanclo et al., 2024). Rana et al. (2020) indicate that atmospheric cold plasma (DBD) improves the in-package shelf life of strawberries, enhancing levels of gallic acid, phloretin, rutin, and total phenolic compounds (Rana et al., 2020) (Table 2).

**Table 2:** Impact of various Cold Plasma types on bioactive compounds.

Class	Polyphenol compound	Food commodity	Plasma type	Treatment conditions	Impact on polyphenol compound	Mechanism of improvement	Reference
Phenolic compound	Gallic acid	Green tea	Nitrogen DBD cold plasma	15 W/ 15 min	Increased	The degradation of EGCG to gallic acid	Keshavarzi et al., 2020
		Strawberry	ACP (DBD system)	60 kV/ 50 Hz/ 15 min	Increased	–	Rana et al., 2020
	Catechin	Green tea	Nitrogen DBD cold plasma	15 W/ 15 min	Increased	–	Keshavarzi et al., 2020
		Pomegranate juice	The cold atmospheric plasma jet (Argon)	5 min/ 4 cm3/0.75 dm3 min_1	Increased	Breakdown of the cell membrane,hydrolysis and depolymerization caused by reactive plasma species.	Herceg et al., 2016
		Pink Lady apples	DBD	12.7 kHz/ 20 min/ 150 W	Increased	–	Tappi et al., 2018
	Epicatechin	Green tea	Nitrogen DBD cold plasma	15 W for 15 min 15	Decreased	–	Dantas et al. (2021)
		Pink Lady apples	DBD	12.7 kHz/ 20 min/ 150 W	Increased	–	Tappi et al., 2018
	Protocatechuic acid	Pomegranate juice	The cold atmospheric plasma jet (Argon)	5 min/ 4 cm3/0.75 dm3 min_1	Decreased		Herceg et al., 2016
	Epigallocatechin gallate (EGCG)	Green tea	Nitrogen DBD cold plasma	15 W/ 15 min	Decreased	The degradation of EGCG to gallic acid	Keshavarzi et al., 2020
	Epicatechin gallate	Açaí pulp	ACP (DBD)	50 Hz/10 min	Increased		Dantas et al., 2021
	*P*-coumaric acid	Açaí pulp	ACP (DBD)	50 Hz/10 min	Increased	An increase in reactive oxygen species in the fruit pulp triggered the activation of the phenylpropanoid pathway, resulting in the production of caffeic acid and p-coumaric acid.	Dantas et al., 2021
	Ferulic acid	Pomegranate juice	The cold atmospheric plasma jet (Argon)	5 min/ 4 cm3/0.75 dm3 min_1	Increased	Breakdown of the cell membrane,hydrolysis and depolymerization caused by reactive species of plasma.	Herceg et al., 2016
	Caffeic acid	Açaí pulp	ACP (DBD) system	50 Hz/10 min	Increased	An increase in reactive oxygen species in the fruit pulp triggered the activation of the phenylpropanoid pathway, resulting in the production of caffeic acid and p-coumaric acid. / The reaction of p-coumaric acid with hydroxyl radicals increases caffeic acid levels.	Dantas et al., 2021
		Pomegranate juice	The cold atmospheric plasma jet (Argon)	5 min/4 cm3/ 0.75 dm3 min_1	Decreased	The capacity of phenolic compounds to scavenge free radicals produced by cold plasma.	Herceg et al., 2016
	Ellagic acid	Pomegranate juice	The cold atmospheric plasma jet (Argon)	5 min/ 4 cm3/0.75 dm3 min_1	Increased	Ellagitannins Hydrolysis to ellagic acid	Herceg et al., 2016
	Hydroxycinnamic acids	Chokeberry juice	cold atmospheric gas phase plasma jet(argon)	25 kHz/ 4 W	Increased	Reacting with radicals that was generated by the plasma	Kovacˇevic´ et al., 2016

Total phenolic acid		Pink Lady apples	DBD	12.7 kHz/10 min/ 150 W	Increased		Tappi et al., 2018
		Strawberry	ACP (DBD system)	60 kV/ 50 Hz/ 15 min	Increased		Rana et al., 2020
		Green tea	Nitrogen DBD atmospheric cold plasma	15 W/ 15 min	Increased	Ion bombardment ruptured the membrane-bound phenolic compounds in the CP, facilitating their extraction.	Keshavarzi et al., 2020
		Mango	Low-pressure atmospheric cold plasma (oxygen)	90 kV/1.4 mbar/5, 10 min	Increased	Minimal processing of fresh produce can increase the release of bound phenolic compounds.	Yanclo et al., 2024
		Pomegranate juice	The cold atmospheric plasma jet (Argon)	5 min/ 4 cm3/0.75 dm3 min_1	Increased		Herceg et al., 2016

## 5 Plasma-activated water

Plasma-activated water (PAW) has been investigated as an alternative method for food processing which can be created by exposing water to plasma from a device either under or above the water's surface. This process generates numerous reactive species and changes in the conductivity and redox potential, which creates an acidic environment (Astorga et al., 2022). Tap water, distilled water, or pure water can undergo electrical discharge treatment to produce PAW. The outcome of this process includes ROS and RNS formed through intricate reactions. Furthermore, interactions between positive and negative compounds with water can lead to the generation of additional radicals (Esua et al., 2020; Gao et al., 2022; Zhou et al., 2018). The food matrix can undergo changes in physical and chemical properties during PAW treatment due to the interaction between ROS and RNS. This interaction alters antioxidant activity, total phenolic compounds, vitamins, and moisture content, as well as affecting structural and textural properties, and certain sensory attributes like color, aroma, and taste. The PAW is generated by various CP reaction systems like plasma jet, corona discharge, surface micro discharge, dielectric barrier discharge, and gliding arc discharge, with DBD and the plasma jet being more prevalent in the food industry (Patra et al., 2022).

### 5.1 Effect of PAW on bioactive compounds

The impact of PAW treatment on polyphenols in fresh-cut pears was investigated across three conditions (peak voltage = 6, 8, and 10 kV) for 5 min. It was observed that the total polyphenol content (TPC) of fresh-cut pears significantly rose (*P* < 0.05) with both the 6-kV and 10-kV PAW treatments within the initial 6 days of storage. However, the beneficial effects disappeared after 8–12 days of storage. This could be attributed to oxidative damage caused by excessive ROS generated during prolonged storage, leading to the degradation of phenolic compounds (determine the effect of PAL and ROS) (Chen et al., 2019). The predominant phenolic profile of rocket leaves was found to be quercetin and quercetin glycosides (approximately 57 % of the total) and during 2, 5, 10, and 20 min treatments a general increase in the overall phenolic content of rocket across all treatments periods. Also, a significant increase was shown in phenolic compounds such as Ferulic acid, Caffeic acid, and Coumaric acid in PW-20. However, 3,5-Dicaffeoylquinic acid in all treatments significantly decreased (Abouelenein et al., 2022). As shown in Table 3, the investigation of sweet orange (*Citrus sinensis*) peel powder revealed an increase in total phenols and total flavonoids when treated with a microbubble PAW reactor under the conditions of 10 kV for 1 h soaking and 10 kV for 2 h soaking. However, total phenols and total flavonoid compounds are enhanced with increasing soaking time (Bansode et al., 2024). The reactive species in PAW, such as RON and ROS, may contribute to disrupting cell walls and assisting in the release of polyphenols, which can be beneficial for increasing polyphenols (Bansode et al., 2024).

**Table 3:** Impact of various Plasma-Activated water types on bioactive compounds.

Class	Polyphenol compound	Food commodity	Plasma type	Treatment conditions	Impact on polyphenol compounds	Reference
Total Phenolic Content		Fresh-cut pears	PAW	6, 10 kV/ 5 min	Increased	Chen et al., 2019
		Tofu	PAW	2 mm hole nozzle/ gas flow rate of 10 L/min/ 2 kV/1 W/ 15 min	Increased up to 80 %	Frías et al., 2020
		Rocket- salad leaves	PAW (Generate by Corona Discharge)	9 kV /5 kHz / 20 min	Increased	Abouelenein et al., 2022
		Sweet orange (*Citrus sinensis*) peel powder	microbubble PAW reactor	10 kV/ 1 and 2 h soaking)	Increased	Bansode et al., 2024

Phenolic acid	Ferulic acid	Rocket- salad leaves	PAW (Generate by Corona Discharge	9 kV /5 kHz /20 min	Increased	Abouelenein et al., 2022
	Caffeic acid	Rocket- salad leaves	PAW (Generate by Corona Discharge	9 kV /5 kHz /20 min	Increased	Abouelenein et al., 2022
	Coumaric acid	Rocket- salad leaves	PAW (Generate by Corona Discharge	9 kV /5 kHz / 20 min	Increased	Abouelenein et al., 2022
	Chlorogenic acid	Rocket- salad leaves	PAW (Generate by Corona Discharge	9 kV /5 kHz /2, 5 min	Increased	Abouelenein et al., 2022
	3,5-Dicaffeoylquinic acid	Rocket- salad leaves	PAW (Generate by Corona Discharge	9 kV /5 kHz/2, 5, 10, 20 min	Decreased	Abouelenein et al., 2022

Flavonoids compounds	Anthocyanin content	Grapes	PAW (generate by plasma jet)	30 min	Not have significant	Guo et al., 2017

Total flavonoids		Sweet orange (*Citrus sinensis*) peel powder	Microbubble PAW reactor	10 kV/1 and 2 h soaking	Increased	Bansode et al., 2024

## 6 Cold plasma effects on food pigments

Natural pigments such as anthocyanins, carotenoids, betalain, and chlorophyll *a*re highlighted by researchers for their health benefits and bioactive compounds. They might decrease cholesterol synthesis, enhance low-density lipoprotein (LDL) degradation, decrease the thickness of blood vessel walls, lower the risk of myocardial infarction, and prevent cardiovascular diseases (CVD). In addition, these pigments can be utilized in food formulations as natural dyes and antioxidants that enhance quality and stability during storage by inhibiting or delaying oxidation reactions (Amorim et al., 2023; Sharma et al., 2021). On the other hand, the visual appeal significantly influences modern consumers' food choices, with color being a crucial element of food and beverages, this attribute is related to the presence of natural pigments in the food matrix. The characteristic color of food is caused by the delocalization of the electron system which occurs by the chromophores contained in the molecules of natural pigments when they capture light energy. Any negative changes in a product's color due to improper processing or chemical reactions directly affect consumer acceptance (Amorim et al., 2023; Dey & Nagababu, 2022). Thus, producers must ensure they use appropriate processing technology.

### 6.1 Anthocyanins

Anthocyanidins are a group of phytochemicals, natural pigments that give color (blue, red, pink, purple, and orange) to many fruits and vegetables (Ejiofor & Igbokwe, 2021). There are over 500 different anthocyanidins, which vary based on the number and position of hydroxyl and methoxyl groups. The most common anthocyanidins found in fruits and vegetables include cyanidin, pelargonidin, delphinidin, malvidin, petunidin, and peonidin (Ejiofor & Igbokwe, 2021).

Anthocyanins are part of the flavonoid family, characterized by a structure comprising two aromatic rings (A and B) connected by a heterocyclic ring (C) containing oxygen. Typically, anthocyanidins are glycosylated anthocyanins, water-soluble vacuolar pigments whose expression and intensity vary with pH and structural composition. They are derived from flavonols and feature the basic structure of the flavylium ion, lacking ketone oxygen at the 4-position. The conjugated bonds in anthocyanins produce red, blue, and purple colors. Anthocyanins are classified based on the placement and number of hydroxyl and methoxyl groups as substituents in the flavylium structure. There are over 23 distinct types of anthocyanidins, with cyanidin, pelargonidin, delphinidin, peonidin, petunidin, and malvidin being the most common (Amorim et al., 2023; Kumar et al., 2023; Sharma et al., 2021). Several studies indicated an increase in anthocyanin contents after treatments such as sour cherry Marasca juice (Garofulić et al., 2015), pomegranate juice (Kovačević, Putnik, et al., 2016), blueberries (Ji et al., 2020; Zhou et al., 2023).

A study by Kovačević, Kljusurić, et al. (2016) found that the optimal cold plasma treatment for pomegranate juice to maximize anthocyanin stability involved a 3-min duration, a 5 cm^3^ sample volume, and a gas flow of 0.75 dm^3^/min, which increased anthocyanin content by 21–35 % (Kovačević, Putnik, et al., 2016). In contrast, their research on chokeberry juice revealed a 23 % loss of anthocyanins during plasma jet treatment. This increase was attributed to enhanced extractability and disruption of cell membrane integrity in the juice's cloudy particles, as well as improved chemical stability through better interactions between phenols and their glycosylated forms (such as glycosylated delphinidin and cyanidin). The study also noted that glycolized compounds like quercetin-4’-O-monoglucoside, quercetin-3,4’-O-diglucoside, and rutin degraded more slowly than their aglycon counterparts. Also, they reported, that increasing gas flow and sample volume could further elevate total anthocyanin content (Kovačević, Kljusurić, et al., 2016).

Mehta et al. (2019) studied the effect of alternating current plasma (ACP) processing on tomato-based beverages. They found that optimal anthocyanin levels were achieved with a gas flow of 50 mL/min for 15 min, resulting in maximum a* (redness) values due to increased polyphenols and anthocyanins. Additionally, ACP promotes co-pigmentation, enhancing the stability of anthocyanins and inducing color variations through chromophore groups (Mehta et al., 2019).

The study by Hou et al. (2019) on blueberry juice treatment with Jet cold plasma found that the optimal treatment duration was 2 min at 0.5 % oxygen concentration. The experiment varied time and oxygen levels, revealing that increased oxygen concentration and prolonged exposure decreased anthocyanins due to oxidative degradation from plasma-generated reactive particles. This sensitivity of anthocyanins to temperature, pH, oxygen, light, and metal ions was noted (Hou et al., 2019). In addition, some researchers have confirmed that cold plasma effectively increases anthocyanin levels in blueberries (Ji et al., 2020; Zhou et al., 2023).

In addition, a study by Poomanee et al. (2021) demonstrated that low-pressure plasma treatment enhances anthocyanin extraction from Luem Pua rice pericarp. This treatment increases water absorption through the rough surface, resulting in a higher anthocyanin content and improved antioxidant properties than untreated rice (Poomanee et al., 2021). Recent research on Pin-to-plate cold plasma bioactive extraction from butterfly pea flowers revealed that applying plasma at 180 V for 5 min increases anthocyanin content in the extracts by 92 %, making it a sustainable and environmentally friendly method Table 4 (Jan & Gavahian, 2024).

**Table 4:** Cold plasma effect on Anthocyanins, Carotenoids, Chlorophyll, and Betalains in the food matrix.

Food matrix	Pigment	Experimental condition					Process effects	Reference
		Plasma source	Power/ Voltage	Gas flow	Exposure time	Frequency		
Sour cherry Marasca juice	Anthocyanins	Cold atmospheric pressure gas phase plasma (plasma jet) Gas; argon	4 W 2.5 kV		3 min	25 kHz	Anthocyanins increased.	Garofulić et al., 2015
Pomegranate juice	Anthocyanins	Cold atmospheric gas phase plasma (plasma jet) Gas: argon	4 W	0.75 dm^3^ min^_^	3 min 5 min	25 kHz	• The yield increase 21–35 %. Highest concentration of delphinidin-3-glucoside and pelargonidin-3,5-diglucoside. Highest concentrations of Gcyanidin-3-glucoside.	Kovačević, Putnik, et al., 2016
Chokeberry	Anthocyanins	Cold atmospheric gas phase plasma (plasma jet) Gas: argon	4 W	0.75 dm^3^ min^_^	5 min	25 kHz	• 23 % loss of anthocyanins.	Kovačević, Putnik, et al., 2016
Tomato-based beverages	Anthocyanins	Cold atmospheric gas phase plasma		50 mL/min	15 min		• Optimize the contents of anthocyanin maximum a* (redness) values.	Mehta et al., 2019
Blueberry juice	Anthocyanins	Jet cold plasma	11 kV	1.0 L/min oxygen concentration 0, 0.5 %	2 min	1000 Hz	• Increase in anthocyanin content.	Hou et al., 2019
Blueberries	Anthocyanins	Atmospheric pressure plasma (a diffuse coplanar surface barrier discharge 400)	300 W		5,10 min		• No significant change appeared.	Pathak et al., 2020
Camu-camu juice	Anthocyanins	DBD	24 kV		15 min	960 Hz	• Decrease in all processing frequencies 200, 420, 582, 698, and 960 Hz. Lowest in 960 Hz.	de Castro et al., 2020
blueberries	Anthocyanins	ACP (DBD) Gas: N_2_	12 kV		60s	5 kHz	• A significant increase in anthocyanins. Maximum increase of 45 % at day 20.	Ji et al., 2020
Purple glutinous rice	Anthocyanins	Low-pressure plasma (pressure: 6 Torr	100 W		20 min	13.56 MHz	• Enhancing the extract of anthocyanins. Major anthocyanins are Cyanidin-3-O-glucoside.	Poomanee et al., 2021
Açai pulp	Anthocyanins	ACP DBD	20 kV		5, 15 min	500 Hz	• The anthocyanin contents were similar to the untreated sample.	Dantas et al., 2021
Blueberries	Anthocyanins	DBD	45kv		50s		• Anthocyanins Increased.	Zhou et al., 2023
Blue pea flower	Anthocyanins	Pin-to-plate cold plasma	180 V		5 min		• Enhancing extracts by 92 %.	Jan & Gavahian, 2024
Siriguela Juice (purple mombin)	Total carotenoids *ꞵ*-carotene	Glow discharge plasma Gas: N_2_	80 W	10 mL/min	8–12 min	50 kHz	• Increased 30 %. Increased 50 %.	Paixão et al., 2019
Avocado pulp	Carotenoids	DBD		10 mL/min	10 min		• 86 % increase in the carotenoid content compared to the control.	Batista et al., 2021
Carrot juice	Total carotenoids *ꞵ*-carotene		80kv 70kv		4 min 3 min		• Increased carotenoid level.	Amorim et al., 2023
Wolfberries	Carotenoids	DBD	13.64 kV		70 s	2.7 kHz	High levels of carotenoids.	Du et al., 2024
Tomato	Chlorophyll Carotenoid	ACP	60 kV		5 min		• Inhibiting chlorophyll degradation, carotenoid accumulation.	Jia et al., 2022
*Hordeum vulgare*	Chlorophyll	Cold Atmospheric Plasma-Treated Water			20 min		• A higher content of chlorophyll.	Bussmann et al., 2023
Persian lime fruit	Chlorophyll Carotenoid	Cold atmospheric gliding arc plasma			60s		• No change in chlorophyll and carotenoid.	Akaber et al., 2024
Okra pods	Chlorophyll alpha Chlorophyll beta	Cold plasma			30s		• Reduced the content of lycopene and chlorophyll alpha an increase in chlorophyll beta content.	Zielinska et al., 2022
Kiwifruit juice	Chlorophyll a, b Carotenoidcontent	Cold plasma treatment DBD	30 kV		6.7 min		• Slight decrease in chlorophyll a, b 29.60 %, 8.36 % respectively slight decrease 4.17 %.	Kumar, Pipliya, Srivastav, & Srivastava, 2024
Beetroot juice	*Betalains*	Cold atmospheric pressure plasma (CAPP)					Slightly decreased, i.e., by 7.5 % for FLC-dc-APGD and FL*E*-pm-rf-APG systems and 15 % for the FLA-dc-APGD system.	Dzimitrowicz et al., 2021

### 6.2 Carotenoids

Carotenoids are hydrophobic, fat-soluble pigments that appear yellow, orange, or red. They are found in crystalline form within chloroplasts and chromoplasts, primarily associated with proteins and function as accessory pigments in photosynthesis. Over 700 carotenoids have been naturally identified, including β-carotene, α-carotene, β-cryptoxanthin, lycopene, lutein, and zeaxanthin, which are key dietary carotenoids. Carotenoids can bind to two multi-protein complexes in photosystem I (PSI, specifically plastocyanin–ferredoxin oxidoreductase) and photosystem II (a water-plastoquinone oxidoreductase multisubunit complex) (Sharma et al., 2021). Various factors can affect the Carotenoids such as high temperature, high light, the presence of ROS, and acidic pH.

Batista et al. (2021) found that a 10 mL/min gas flow resulted in an 86 % increase in carotenoid content and a 51 % increase in DPPH during the treatment. These short treatments, lasting only 10 min, likely minimized the potential degradation of carotenoids in the pulp by reducing exposure to radicals (Batista et al., 2021). Also, in Siriguela juice total carotenoids and *ꞵ*-carotene increased 30 % and 50 % respectively (Paixão et al., 2019). However, these amounts not change in Persian lime fruit (Akaber et al., 2024) and decreased in kiwifruit juice (Kumar, Pipliya, Srivastav, Srivastava, Battula, & Sen, 2024).

CP is known for producing various reactive species, especially ROS (Hu et al., 2021). These reactive species can break down crystalline carotenoid and pectin complexes in the fruit cell wall, releasing bound conjugated carotenoids, which results in color changes. After treatment, this release can increase carotenoid yield (Ramezan et al., 2024). However, CP treatment lowers pH, which may denature pigment-protein complexes and reduce carotenoid content due to carotenoids' sensitivity to acidic conditions over time. Supramolecular structures in the carotenoid-protein complex protect carotenoids during CP treatment; however, the breakdown by active chemical species of CP diminishes this protective effect (Pant et al., 2022). Therefore, the duration of exposure is crucial for effective carotenoid treatment. In a previous study on wolfberries, the optimal DBD treatment conditions for enhancing wolfberries were a voltage of 13.64 kV, a duration of 70 s, and a frequency of 2.7 kHz. Additionally, moderate DBD treatment can promote flavonoid biosynthesis (Du et al., 2024). A study by Amorim et al. (2023) indicates that treating carrot juice with cold plasma positively affects carotenoid retention, particularly at higher applied voltages. This effect likely arises from the interaction between reactive species generated by the plasma and the plant material, which influences carotenoid release. Cold plasma appears to disrupt chloroplast cell membranes, facilitating the release of pigments and increasing their concentration in the extracellular medium (Amorim et al., 2023). In untreated carrot juice, some carotenoids were likely trapped within suspended carrot particles, but post-treatment, the carotenoid molecules migrated more readily into the liquid, accounting for the observed increase in carotenoid content (Amorim et al., 2023).

### 6.3 Betalain

Betalains are produced by plants in the order Centrospermae (Caryophyllales), such as pitaya (*Hylocereus undatus*), beetroot (*Beta vulgaris* L.), amaranth (Amaranthus), and prickly pear (*Opuntia ficus-indica*). These red-colored vegetables are rich in betalains, comprising red-violet betacyanins (mainly betanin) and yellow-orange betaxanthins (including indicaxanthin and vulgaxanthin). Betalains are water-soluble nitrogen pigments known for their apoptosis and antioxidant properties. Their antioxidant activity increases with hydroxyl and imino groups and decreases with the glycosylation of aglycones. The plasma concentration of betalains effectively promotes their assimilation into lipoproteins and red blood cells, thus protecting against oxidative stress and hemolysis (Amorim et al., 2023; Sharma et al., 2021). Cold plasma reactive species, such as nitric oxide and nitrogen dioxide, can react with moisture in food to form acidic compounds. Betalain is stable within a pH range of 3–7. A decrease in pH may lead to epimerization and deglycosylation, resulting in a color change from red to violet, which is associated with the recondensation of betalamic acid and cyclo Dopa 5-O-glucoside (Calva-Estrada et al., 2022; Ramezan et al., 2024). Dzimitrowicz et al. (2021) investigated the effects of cold atmospheric pressure plasma (CAPP) using three systems: FLC-dc-APGD (direct current atmospheric pressure glow discharge with BRJ as a cathode), FLA-dc-APGD (with BRJ as an anode), and FLE-pm-rf-APGD (pulse-modulated radiofrequency alternating current with BRJ as an electrode). They found that different treatments led to a slight decrease in betalains—7.5 % for FLC-dc-APGD and FLE-pm-rf-APGD, and 15 % for FLA-dc-APGD—due to sensitivity to environmental conditions, pH, and temperature, which may alter their structure and convert them to dehydrogenated forms. Color loss is also inevitable under these conditions (Dzimitrowicz et al., 2021).

### 6.4 Chlorophyll

Chlorophylls are the most abundant natural pigments in plants, composed of a porphyrin head that four pyrrole rings with the help of nitrogen and a long hydrocar- bon tailand are attached to a centrally located magnesium ion. These pigments give plants their green color. Chlorophylls exist in five types (a, b, c, d, f) in two main forms, a and b, commonly found in a 3:1 ratio in fruits and vegetables (Sharma et al., 2021). Chlorophylls are influenced by excess light, heat, acidic conditions (especially with oxygen), enzyme activity, ROS, and ultraviolet (UV) radiation generated by cold plasma. Prolonged heating in an acidic medium causes chlorophylls to convert into pheophytin by replacing the Mg2+ ion in the porphyrin ring with H+. Various factors, including time, frequency, processing gas, and voltage, can also affect chlorophyll concentration (Jurić et al., 2020). A study on postharvest tomatoes indicated that the red color development during storage decreased from 80 % in the control group to 40 % in the treatment group exposed to 60 kV Atmospheric Cold Plasma (ACP) for 5 min. This treatment helps maintain the green color of postharvest tomatoes by regulating chlorophyll metabolism and delaying chlorophyll degradation. Chlorophyllase and chlorophyll oxidase degrade chlorophyll into dephytochlorophyll, leading to the formation of coloring pigments like carotenoids, which contribute to the redness of tomatoes. Carotenoids accumulate less in the early stages of ripening but increase rapidly in the later stages (Jia et al., 2022).

A study by Bussmann et al. (2023) on treating barley leaves (*Hordeum vulgare* cv. Kosmos) with Plasma-treated water (PTW) found that PTW treatment increased chlorophyll content, quantum yield, and total ascorbate levels in the leaves. The system used a pin-to-liquid discharge configuration with four metal electrodes positioned about 3 mm from the 900 mL water surface for 20 min (Bussmann et al., 2023). The study on Persian lime fruit treated with cold atmospheric gliding arc plasma showed no changes in chlorophyll or carotenoid levels (Akaber et al., 2024). Also, the study on chlorophyll *a*lpha and chlorophyll beta indicated a decrease and increase respectively in okra pods (Zielinska et al., 2022). Kumar, Pipliya, Srivastav, and Srivastava (2024) research on kiwifruit juice showed chlorophyll is more heat-sensitive than carotenoids. They observed slightly decreased chlorophyll a, b, and carotenoid content during cold plasma treatment. Their findings also indicated that the optimal cold plasma conditions, which resulted in minimal changes in the ΔE values of the kiwifruit juice, were 30 kV/5 mm/6.7 min (Kumar, Pipliya, Srivastav, Srivastava, Battula, & Sen, 2024). Research on camu-camu juice revealed that DBD cold plasma reduced levels of compounds at all processing frequencies: 200, 420, 582, 698, and 960 Hz, with the lowest levels observed at 960 Hz. Higher excitation frequencies increased the energy density of plasma ions and electrons, leading to greater production of ozone and free radicals. These reactive species interacted with anthocyanin molecular structures, resulting in glycosylated chalcones, which were then cleaved, rapidly degrading into phenolic acids and aldehydes (de Castro et al., 2020).

### 6.5 Cold plasma effects on synthetic pigments

Azo dyes are the most abundantly produced dyes (over 60 %) and their importance is likely to grow. They are widely used in the food, pharmaceutical, cosmetics, paper, textile, and leather industries (Barciela et al., 2023). Hafeez et al. (2021) investigated the degradation of the benzidine-based azo dye, Reactive Black-5 (RB-5), as a model compound using a non-thermal hybrid corona-DBD plasma micro-reactor. Dye removal studies suggest ozone primarily attacks chromophoric groups, like azo groups (N=N) or C=C bonds connecting aromatic rings. Ozone favors the decomposition of amino or azo groups (carcinogenic components), leading to dye discoloration through chromophore breakdown. The degradation of RB-5 by ozone begins with sulfonic group detachment and azo bond cleavage, accompanied by the introduction of hydroxyl (OH) groups generated during oxidation. Subsequent reactions involve further sulfonic group detachment, azo bond cleavage, and hydroxylation, producing naphthalene and alkylsulfonyl phenolic intermediates. Ultimately, hydroxylation and sulfonic reactions result in smaller organic molecules, such as CO2, H2O, and acetic acid. A detailed ozonation pathway for RB-5 degradation has been established (Hafeez et al., 2021).

## 7 Cold plasma effects on enzyme

Browning in fruits and vegetables results from both enzymatic and nonenzymatic processes. Enzymatic browning is driven by peroxidases and polyphenol oxidases, while nonenzymatic browning occurs through the degradation of ascorbic acid and the formation of polyphenol o-quinones (Bayati et al., 2024). PPO and peroxidase (POD) are key enzymes involved in fruit spoilage. Studies on various fruits have shown that cold plasma treatment effectively inactivates these enzymes Table 5 (Dantas et al., 2021; de Castro et al., 2020; Kumar, Pipliya, Srivastav, & Srivastava, 2024; Paixão et al., 2019). This mechanism relates to the structure of food enzymes, which are composed of primarily proteins and complex proteins. The cold plasma inactivation mechanism involves the cleavage of specific bonds by active species, leading to chemical modifications in side chains and resulting in the loss of the enzymes' secondary protein structure. The side chains may include cysteine and the aromatic rings of phenylalanine, tyrosine, and tryptophan (Sruthi et al., 2022). However, Ji et al. (2020) found that a brief 60-s treatment at 12 kV increased the activities of PPO, SOD, and catalase without affecting POD (Ji et al., 2020).

**Table 5:** Cold plasma effects on different enzymes.

Enzyme	Food matrix	Plasma source	Experimental condition				Process effects	Reference
			Power/Voltage	Frequency	Exposure time	Other		
Superoxide dismutase	Blueberries	Atmospheric cold plasma DBD Gas: N_2_	12 kV	5 kHz	60s		• 15.7 % higher than the control group at 20 days	Ji et al., 2020
Polyphenol oxidase (PPO)	Siriguela Juice (purple mombin)	Glow discharge plasma Gas: N_2_	80 W	50 kHz	15 min	20 mL/min	• Reducing 20 %	Paixão et al., 2019
	Blueberries	ACP DBD Gas: N_2_	12 kV	5 kHz	60s		• Increased by up to 14 %	Ji et al., 2020
	Camu-camu juice	DBD	24 kV	960 Hz	15 min	40 mL	• Enzyme inactivation	de Castro et al., 2020
	açai pulp	ACP DBD	20 kV	50 Hz	15 min		• Inactivating polyphenol oxidase (82.4 %)	Dantas et al., 2021
	Kiwifruit juice	DBD	24 kV 30 kV 36 kV		8.71 min 4.41 min 2.51 min	Juice depth6 mm	• Inactivation 71.29 %	Kumar, Pipliya, Srivastav, Srivastava, Battula, & Sen, 2024
Catalase	Blueberries	ACP DBD Gas: N_2_	12 kV	5 kHz	60s		• Significantly higher than that of the control group	Ji et al., 2020
Peroxidase (POD)	Siriguela Juice (purple mombin)	Glow discharge plasma Gas: N_2_	80 W	50 kHz	15 min	20 mL/min	• Reducing 6 %	Paixão et al., 2019
	Blueberries	ACP DBD Gas: N_2_	12 k	5 kHz	60s		• No immediate effect on it but accelerated it after 20 days.	Ji et al., 2020
	Camu-camu juice	DBD	24 kV	960 Hz	15 min	40 mL	• Enzyme inactivation	de Castro et al., 2020
	Açai pulp	ACP DBD	20 kV	50 Hz	15 min	The açaí pulp placed:30 mL	• Inactivating peroxidase (42.3 %)	Dantas et al., 2021
	Kiwifruit juice	DBD	24 kV 30 kV 36 kV		11.37 min 5.22 min 3.60 min	juice depth6 mm	• Inactivation 61.45 %	Kumar, Pipliya, Srivastav, & Srivastava, 2024
Dihydroflavonol 4-reductase (DFR)		DBD-CP	45 kV		50s		• Increased the activities	Zhou et al., 2023
Anthocyanidin synthase (ANS)		DBD-CP	45 kV		50s		• Increased the activities	Zhou et al., 2023
Flavonoid 3-O-glucosyltransferase (UFGT)		DBD-CP	45 kV		50s		• Increased the activities	Zhou et al., 2023
Tyrosinase		Low-pressure plasma (pressure: 6 Torr)	100 W	13.56 MHz	20 min		• Anti-tyrosinase activity	Poomanee et al., 2021
Lipoxygenase		DBD	70 V	60 Hz	5-10 min		• Decreased the activities	Zhao et al., 2024
Lipolytic enzyme (lipase, phospholipase)		DBD	70 V	60 Hz	5-10 min		• Decreased the activities	Zhao et al., 2024

Additionally, Kumar, Pipliya, Srivastav, and Srivastava (2024) reported that POD is more resistant to cold plasma than PPO during inactivation (Kumar, Pipliya, Srivastav, & Srivastava, 2024). In blueberries, cold plasma treatment significantly enhanced the activities of dihydroflavonol 4-reductase (DFR), anthocyanidin synthase (ANS), and flavonoid 3-O-glucosyltransferase (UFGT), particularly DFR and UFGT, which are vital for metabolizing phenols, flavonoids, and anthocyanins in the phenylpropanoid pathway (Zhou et al., 2023). Poomanee et al. (2021) demonstrated that cold plasma treatment inhibits tyrosinase in purple glutinous rice, an enzyme responsible for browning in damaged fruits and plant tissues (Choma & Nikolaichuk, 2022; Poomanee et al., 2021).

## 8 Cold plasma effects on vitamins

Vitamin C or Ascorbic acid is a water-soluble vitamin with a high antioxidant capacity. Fresh berries are a good source of this vitamin. This vitamin can decay through deprotonation, resulting in dehydroascorbate. Factors that accelerate this process include hydrogen peroxide, tocopheroxyl radical, and superoxide. Zhou et al. (2023) and Ji et al. (2020) found that treating blueberries with DBD cold plasma reduced ascorbic acid levels (Ji et al., 2020; Zhou et al., 2023). However, Ji et al. (2020) showed that after 10 days, ascorbic acid levels in treated samples surpassed those in the control. This may be due to active species produced by cold plasma, which enhances the activity of dehydroascorbic acid reductase, resulting in a higher production rate of ascorbic acid compared to its oxidation rate. Consequently, the ascorbic acid content in ACP-treated blueberries declines more slowly than in the control group. However, Pathak et al. (2020) reported not have significant change in ascorbic acid in blueberries (Pathak et al., 2020). Kiwifruit juice showed a 9 % reduction during treatment, likely due to the degradation of ascorbic acid from oxidizing species, such as ozone, generated in the chemical process. It is hypothesized that this degradation occurs through a direct interaction with ozone via a Criegee mechanism or an indirect process involving excited molecules and singlet oxygen mediated by free radicals (Kumar, Pipliya, Srivastav, Srivastava, Battula, & Sen, 2024). The study on Siriguela juice found that glow plasma treatments increased vitamins A, B3, and B6 (Paixão et al., 2019). The boost in vitamin A observed in acerola juice (Fernandes et al., 2019) and Siriguela juice (Paixão et al., 2019) may be linked to the disruption of lipoprotein bonds, facilitating its release Table 6.

**Table 6:** Cold plasma effects on different vitamins.

Vitamin	Food matrix	Plasma source	Experimental condition				Process effects	Reference
			Power/Voltage	Frequency	Exposure time	Other		
Ascorbic acid	Blueberries	ACP DBD Gas: N_2_	12 kV	5 kHz	60s		• 7.7 % reduced after treatment	Ji et al., 2020
	Blueberries	Atmospheric pressure plasma (a diffuse coplanar surface barrier discharge 400) Gas: N_2_	300 W		5,10 min		• Not have a significant change	Pathak et al., 2020
	Blueberries	DBD-CP	45 kV		50 s		• Decreased the level of ascorbic acids	Zhou et al., 2023
	Kiwifruit juice	CP DBD	30 kV		6.7 min	5 mm	• Decreased 9 %	Kumar, Pipliya, Srivastav, & Srivastava, 2024
	Camu-camu juice	DBD	24 kV	960 Hz	15 min	40 mL	• Increased vitamin C concentration	de Castro et al., 2020
	Siriguela Juice (purple mombin)	Glow discharge plasma Gas: N_2_	80 W	50 kHz	(5–15 min)	(10–30 mL/min)	• Not have significant change	Paixão et al., 2019

Vitamin A	Siriguela Juice (purple mombin)	Glow discharge plasma Gas: N_2_	80 W	50 kHz	(8–12 min)	(10 mL/min)	• Increasing 50 %	Paixão et al., 2019
	Acerola juice	Glow plasma treatment Gas: N_2_		80 kHz	10 min		• Increasing	Fernandes et al., 2019

B_3_	Siriguela Juice (purple mombin)	Siriguela Juice (purple mombin)	80 W	50 kHz	10 min	10 mL/min	Increasing 20 %	Paixão et al., 2019

B_6_	Siriguela Juice (purple mombin)	Siriguela Juice (purple mombin)	80 W	50 kHz	10 min	20 mL/min	• Increasing 56 %	Paixão et al., 2019

## 9 Cold plasma effects on lipids

Plasma generates ROS that influence the formation of saturated bonds, which induces lipid oxidation, transforming unsaturated lipids into saturated lipids. This process increases palmitic and stearic acids while reducing oleic, and linoleic acids. Hydroperoxyl radicals, superoxide radicals, and singlet oxygen alter unsaturated fatty acids. Additionally, lipid oxidation affects foods' sensory and nutritional quality, highlighting the need for further studies on this topic (Fernandes & Rodrigues, 2021). The study by Cao et al. (2024) indicated that five fatty acids found in rice—palmitic acid (C16:0), stearic acid (C18:0), oleic acid (C18:1n9c), linoleic acid (C18:2n6c), and α-linolenic acid (C18:3n3)—are affected by cold plasma treatment, which can decrease unsaturated fatty acids while increasing saturated fatty acids with prolonged exposure (Cao et al., 2024).

Sang et al. (2024) indicated that primary lipid oxidation increased following treatment, while palmitic acid levels rose (Sang et al., 2024). In contrast, the levels of stearic acid, oleic, and linoleic acid decreased. Several studies have utilized cold plasma technology to create healthier trans-fat-free margarine from palm oil and investigated this new technology Table 7 (Priyanti et al., 2024; Sirati et al., 2023). Priyanti et al. (2024) observed a decrease in mono-, di-, and tri-unsaturated fatty acids and a corresponding increase in saturated fatty acids when using helium gas and glycerol as a hydrogen source. This is likely due to the higher plasma power, which increased helium dissociation and the concentration of reactive species. These reactive species collided with glycerol, generating hydrogen radicals. The resulting high electron density and energy, typical of surface discharge, were sufficient to break bonds in the plasma-liquid interaction. Metastable helium and electron energy levels then facilitated the addition of Ḣ radicals to C=C bonds, creating stable single bonds. Consequently, the formation of high-energy electrons, ions, and radicals offers a pathway to convert unsaturated fats into saturated fats with low or no trans-fat content. (Priyanti et al., 2024). Another study on palm oil showed that treatment at 50 W for 30 min had a minimal impact (0.2 %) on fatty acids. It was noted that treatment time and power are crucial factors in cold plasma treatment. Increasing the power to 60 W and extending the duration to 45 min resulted in a decrease in unsaturated fatty acids and an increase in saturated fatty acids (Niveditha et al., 2023).

**Table 7:** Cold plasma effects on lipids.

Food matrix	Plasma source	Experimental condition				Process effects	Reference
		Power/Voltage	Frequency	Exposure time	Other		
Rice	low-pressure radiofrequency (RF) cold plasma Gas: helium	120 W	13.56 MHz	20s	140 Pa, 300 g sample	• Palmitic acid (C16:0) not have significant. Stearic acid (C18:0) increased. Oleic acid (C18:1n9c) decreased. Linoleic acid (C18:2n6c) decreased. α-linolenic acid (C18:3n3) decreased.	Cao et al., 2024
Tilapia Fillets	DBD	70 kV		3 min		• Palmitic acid (C16:0) increased. Stearic acid (C18:0) decreased. Oleic acid (C18:1) decreased. Linoleic acid (C18:2n-6) decreased.	Sang et al., 2024
Oyster	DBD	70 V	60 Hz	5-10 min		• Palmitic acid (C16:0) decreased. Stearic acid (C18:0) decreased. Oleic acid (C18:1, n-9) decreased. Eicosapentanoic acid (EPA, C20:5, n-3) decreased. Eicosanoenoic acid (C20:1, n-9) decreased. Docosahexanoic acid (DHA, C22:6, n-3) decreased.	Zhao et al., 2024
Milk	DBD	70 kV		15 min	50 mL	• Palmitic acid (C16:0) increased. Stearic acid (C18:0) increased. Oleic acid (C18:1n9c) not have significant. Butyric acid (C4:0) decreased. Lauric acid (C12:0) decreased. Myristic acid (C14:0) not have significant.	Abbas et al., 2024
Palm oil	Bell-jar type plasma reactor	50 W	13.56 MHz	30 min		• Palmitic acid (C16:0)) not have significant. Stearic acid (C18:0)) not have significant. Oleic acid (C18:1)) not have significant. Linoleic acid (C18:2n-6)) not have significant.	Niveditha et al. (2023)
Palm oil	Parallel-type dielectric barrier discharge (DBD)	75 W		12 h		• Palmitic acid (C16:0) decreased. Oleic acid (C18:1 n-9 *cis*) decreased. Linoleic acid (C18:2 n-6 *cis*) decreased. α-linolenic acid (C18:3 n-3) decreased. Lauric acid (C12:0) increased. Myristic acid (C14:0) increased. Palmitoleic acid (C16:1) increased. Stearic acid (C18:0) increased. C18:1 n-9 *trans* increased.	Priyanti et al., 2024
Soybean oil	Surface dielectric barrier discharges cold plasma (SDBDCP) gas: 100 % hydrogen	15 kV		13 h		• Saturated fatty acids Monounsaturated fatty acids (13.98 %) increased. Polyunsaturated fatty acids (17.64 %) decreased.	Sirati et al., 2023
Sesame (*Sesamum indicum* L.) Sunflower (*Helianthus annuus* L.)	DBD	25 kV	50 Hz	15 min	5 mL/min	• Unsaturated fatty acid content (5 %) increased. Unsaturated fatty acid content (3.9 %) increased.	Afshar et al., 2022

Additionally, in studies of sesame and sunflower oils, treatment for 15 min slightly increased unsaturated fatty acid content, while longer treatment times (30 min) led to an increase in saturated fatty acid content (Afshar et al., 2022).

## 10 Cold plasma effects on essential oils

EOs are aromatic, volatile, colorless compounds with pleasant odors, including hydrocarbons, terpenes, alcohols, aldehydes, and ketones, which help stabilize cellular free radicals. They are derived from various parts of plants such as flowers, roots, bark, leaves, seeds, peels, fruits, and wood. EOs are known for their beneficial effects, including antioxidant, anti-inflammatory, antiviral, and anti-cancer properties. CAPP treatment enhances the synthesis and efficacy of EOs and expands their potential applications (Khan et al., 2024). A study on cumin essential oil extraction showed a 44 % increase in efficiency with cold plasma treatment (DBD) using Ar gas, leading to an increase in cumin aldehyde from 47.9 % to 56.4 % (Shokoohi et al., 2024).

Research by Rezaei et al. (2021) on fennel and spearmint demonstrated that DBD cold plasma could enhance essential oil yield at 19 kV for 10 min. *E*-Anethole, the main component of fennel at 83.9 %, showed a slight increase after 2.93 min at 19 kV but decreased with a longer exposure (Rezaei et al., 2021). Also, Gunathilake et al. (2025) found that both atmospheric and low-pressure cold plasma treatments increased the extraction yield of black pepper's volatile oil and piperine content. While both methods increased the dominant sesquiterpene (caryophyllene), they reduced dominant monoterpenes (Gunathilake et al., 2025).

Overall, cold plasma treatment positively affects extraction yields and volatile oil content across various sources Table 8 (Buonopane et al., 2016; Ebadi et al., 2019; Gunathilake et al., 2025; Hemmati et al., 2021; Karunanithi et al., 2023).

**Table 8:** Cold plasma effects on essential oils.

Food matrix	Essential oil component (EO)	Plasma source	Experimental condition			Process effects	Reference
			Power/ Voltage	Frequency	Exposure time		
Black Pepper Seeds (*Piper nigrum*)	α-pinene β-pinene sabinene caryophyllene d-limonene dominant monoterpenes dominant sesquiterpene (caryophyllene	Low-pressure Cold Plasma treatment (LPCP) gas: normal air 0.22 mbar Atmospheric cold plasma gliding arc plasma discharge (GAPD)	300 W 230 V	13.56 MHz. 50 Hz	20 min 20 min	Decreased Decreased decreased increased decreased decreased increased	Gunathilake et al., 2025
Cumin seeds	cumin aldehyde	argon atmospheric cold plasma (DBD)	17–19 kV		30S	• Increased content (47.9–56.4 %)	Shokoohi et al., 2024
Betel leaf powder	Chavibeto chavibetol acetate hydroxychavicol chavicol eucalyptol isoeugenol acetate eugenol	atmospheric pressure cold plasma+ Microwave treatment gas: oxygen	810 W	50 Hz	90S	• Increased 3.29 % Increased 11.15 % Increased 2.12 % Increased 0.34 % Increased 0.08 % Increased 0.42 % Increased 0.41 %, 3.31 % extraction yield	Karunanithi et al., 2023
Fennel (*Foeniculum vulgare* Mill.)	E-Anethole Fenchone pAllylanisole	DBD	19 kV		10 min	• Decrease 1.4 % Increase 0.4 % Increase 0.3 % Optimize the EOs yields extraction 1.83 (% *v*/*w*)	Rezaei et al., 2021
Spearmint (*Mentha spicata* L.)	Carvone Dihydro carveol Pulegone Limonene Caryophyllene 1,8-cineole	DBD	19 kV		10 min	Decrease 0.4 % Decrease 1.6 % Decrease 1.2 % Increase 3.1 % Decrease 1 % Increase 0.6 % Optimize the EOs yields extraction 1.81 (% v/w)	Rezaei et al., 2021
Turmeric powder	ar-Turmerone α-Zingiberene α-Turmerone β-Turmerone ar-Turmerol β-Sesquiphellandrene	atmospheric-pressure (DBD)	25 kV		7 min	increased approximately 6 % increased approximately 3 % decreased approximately 2 % decreased approximately 2 % decreased approximately 2 % increased approximately 3 %	Hemmati et al., 2021
Lemon verbena	EO content (*V*/*W* %) Citral (geranial + neral) Limonene 1,8-cineole γ-elemene Spathulenol globulol	Low-pressure cold plasma Feeding gases: nitrogen (N2), argon (Ar), and oxygen (O2).	1200 W	2.45 GHz	1 min 3 min 3 min 3 min 3 min 5 min 5 min	• Increased content 36.7 % Decreased content 17.3 % Slightly increased content 7.3 %, Slightly increased content 2.6 %, Slightly increased content 2.9 %, Increased content 2.9 % Increased content 3.6 %	Ebadi et al., 2019
Sweet basil	Eugenol	Plasma jet Feeding gas: helium and air			30s	• Increased antioxidant effect. A greater concentration of eugenol.	Buonopane et al., 2016

## 11 Limitations of cold plasma treatment

The use of plasma technology for modifying the structure of food pigments, bioactive compounds, enzymes, vitamins, fatty acid, and essential oils has shown promise in food science, but it also comes with several limitations. Here are some of the key challenges and limitations associated with using plasma in food modification: 1. Process Control or complexity of Treatment Parameters: The effectiveness of plasma treatment can be highly dependent on various parameters such as the type of plasma (DBD plasma, atmospheric pressure plasma), treatment time, gas composition, and pressure. Optimizing these parameters for different food matrices can be complex and may require extensive experimentation (Leandro et al., 2024). 2. Non-specific Reactions: Plasma treatment may lead to non-specific modifications that can alter multiple components within the food matrix. This lack of specificity can complicate the characterization of treated materials, as it may not be clear which modifications contribute to the desired properties (Amirabadi et al., 2020). 3. Process Scalability: While laboratory-scale experiments with plasma modification may yield promising results, scaling up the process for industrial applications can pose challenges. The design and implementation of large-scale plasma systems can be cost-prohibitive and technically challenging (Leandro et al., 2024). 4. Effects on Nutritional Quality: While plasma treatment can lead to desirable changes such as improved solubility or enhanced functionality, it may also affect the nutritional quality of food components. There is a possibility of degradation of essential nutrients or bioactive compounds, which needs to be carefully evaluated (Amirabadi et al., 2020). 5. Limited Understanding of Mechanisms: The fundamental mechanisms by which plasma modifies carbohydrates, gums, starches, and proteins are not fully understood. This lack of understanding can hinder the ability to predict outcomes and optimize processes effectively (Thirumdas et al., 2015). 6. Consistency and Reproducibility: Achieving consistent and reproducible results can be a challenge, as plasma interactions with food matrices may vary. Variations in the material or experimental setup can lead to inconsistent treatment effects (Basak & Annapure, 2022). 7. Effects on Sensory Properties: Changes in the chemical structure of food components can lead to alterations in flavor, texture, and overall sensory attributes. These changes may not always be desirable and can affect consumer acceptance (Olatunde et al., 2021). 8. Cost Considerations and scalability: The installation and maintenance of plasma systems can be costly, which may limit their widespread adoption in food processing industries, especially for small and medium-sized enterprises(Basak & Annapure, 2022). 9. Regulatory and Safety Concerns: The introduction of plasma-modified ingredients into food products may raise regulatory and safety concerns, requiring thorough assessments and approvals before commercialization (Leandro et al., 2024). 10. Limited Research: While there is growing interest in plasma technology, it is still relatively new in the context of food modification. There is a need for more research to fully explore its potential and limitations, as well as its long-term effects on food quality and safety (Leandro et al., 2024). 11. Limited Penetration Depth: Plasma treatments may not penetrate deeply into bulk materials, leading to uneven modification. This can be particularly problematic for larger particles or dense matrices (Basak & Annapure, 2022). 12. Safety and Toxicity Concerns: The generation of reactive species during plasma treatment raises concerns about potential toxicity or the formation of harmful compounds. Ensuring that treated products are safe for consumption is critical (Thirumdas et al., 2015). 13. Regulatory Challenges: The use of novel technologies like plasma in food processing may face regulatory hurdles. Ensuring compliance with food safety standards and gaining approval for new processes can be time-consuming (Basak & Annapure, 2022). 14. Consumer Acceptance: There may be consumer resistance to foods treated with novel technologies. Educating consumers about the benefits and safety of plasma-modified foods is essential for market acceptance (Leandro et al., 2024). 15. Variability in Food Matrices: Different food matrices can respond differently to plasma treatment due to their unique compositions and structures. This variability can complicate the development of standardized treatment protocols (Olatunde et al., 2021) 16. Degradation Risks: While plasma can enhance certain properties (solubility or emulsification), it may also cause degradation of sensitive components, leading to loss of nutritional value, flavor, or other desirable characteristics (Sruthi et al., 2022). 17. Scale-Up Challenges: While laboratory-scale experiments may show promising results, scaling up plasma treatment for industrial applications can be technically and economically challenging. Equipment costs and energy consumption may be significant barriers (Olatunde et al., 2021). 18. Reproducibility Issues: The effects of cold plasma treatment can be inconsistent, leading to variability in the modification of carbohydrates and proteins. Factors such as gas composition, treatment time, and power levels can all influence the outcomes, making it difficult to achieve uniform results (Sruthi et al., 2022). 19. Chemical Reactions: Cold plasma can induce various chemical reactions, including oxidation, which may lead to undesirable changes in the flavor, color, or texture of food products. This can be particularly concerning in food applications where sensory qualities are critical (Olatunde et al., 2021). Despite these limitations, ongoing research continues to explore ways to optimize cold plasma technology for modifying carbohydrates and proteins, potentially overcoming some of these challenges in the future. In summary, while plasma technology offers promising avenues for modifying food components, careful consideration of these limitations is necessary for effective application in food processing.

## 12 Conclusion

Cold plasma processing is a non-thermal technology that helps maintain food quality. It's essential to examine how cold plasma conditions, contributing factors and reactive species affect various food components and their properties. Bioactive components in foods can be influenced by cold plasma through primary mechanisms like cell membrane disruption. Studies have shown that cold plasma treatment can enhance total phenolic acids and anthocyanins. Additionally, this treatment modifies the secondary structure of enzyme proteins, leading to the inactivation of enzymes such as PPO and POX. Furthermore, cold plasma processing preserves essential oils post-treatment. Therefore, optimizing cold plasma treatment parameters can mitigate negative impacts on quality attributes, preserving bioactive compounds, essential oils, and vitamins, reducing lipid oxidation, and enhancing pigments and sensory characteristics.

## CRediT authorship contribution statement

**Shiva Vedaei:** Writing – original draft, Investigation. **Arash Dara:** Writing – review & editing, Conceptualization.

## Funding

No funding was received.

## Declaration of competing interest

The authors declare that they have no known competing financial interests or personal relationships that could have appeared to influence the work reported in this paper.

## Data Availability

This research is based on a review of data from other researchers.
